# A Cold and Its Treatment

**Published:** 1889-12

**Authors:** 


### A COLD AND ITS TREATMENT.

When exposed to the influence of damp air, sudden changes of temperature, etc., the skin becomes chilled, and the blood is driven from the outside surface to the deeper-seated organs of the body, causing congestion of the mucous membranes lining the air tube and digestive canal, followed by chills, suppressed perspiration, aches and pains in the bones and back, with slight fever, the disease is termed a common cold. Treatment: This disease is best treated by a warm bath at bed-time and ten grains of Dover's Powder, with a free use of warm drinks. Three grains for a child.

